# Experimental and Numerical Study of Downward Flame Spread over Glass-Fiber-Reinforced Epoxy Resin

**DOI:** 10.3390/polym14050911

**Published:** 2022-02-24

**Authors:** Oleg Korobeinichev, Alexander Karpov, Artem Shaklein, Alexander Paletsky, Anatoliy Chernov, Stanislav Trubachev, Roman Glaznev, Andrey Shmakov, Sergey Barbot’ko

**Affiliations:** 1 Voevodsky Institute of Chemical Kinetics and Combustion SB RAS, 630090 Novosibirsk, Russia; paletsky@kinetics.nsc.ru (A.P.); chernov@kinetics.nsc.ru (A.C.); satrubachev@gmail.com (S.T.); r.k.glaznev@gmail.com (R.G.); shmakov@kinetics.nsc.ru (A.S.); 2 Udmurt Federal Research Center, 426067 Izhevsk, Russia; karpov@udman.ru (A.K.); mx.oryx@gmail.com (A.S.); 3 Department of Physics, Novosibirsk State University, 630090 Novosibirsk, Russia; 4 All-Russian Scientific Research Institute of Aviation Materials, 105005 Moscow, Russia; slbarbotko@yandex.ru

**Keywords:** flame spread, opposed flow, polymer composites, numerical modeling, coupled model, temperature measurement, thermal conductivity, glass fiber reinforcement, combustion, pyrolysis

## Abstract

For the first time, a comprehensive study of downward flame spread over glass-fiber-reinforced epoxy resin (GFRER) slabs in oxidizer flow has been carried out experimentally and numerically. Microthermocouples were used to measure the temperature profiles on the solid fuel’s surface and in the flame, and a video camera was used to measure the rate of flame spread (ROS). The ROS was found to be linearly dependent on the oxygen concentration, to be inversely proportional to the slab thickness and not to depend on the direction of the flame spread over the slab. The absence of the influence of the forced oxidizing flow velocity and the weak influence of the GFRER pyrolysis kinetics on the ROS were observed. For the first time, a numerical model of flame spread over reinforced material with thermal conductivity anisotropy was developed on the basis of a coupled ‘gas–solid’ heat and mass transfer model, using modifications of the OpenFOAM open-source code. The sensitivity analysis of the model showed that the thermal conductivity in the normal direction to the GFRER surface had a much greater effect on the ROS than the thermal conductivity along the direction of flame propagation. The numerical results show good agreement with the experimental data on the dependences of the ROS on oxygen concentration, slab thickness and the N_2_/O_2_ mixture flow velocity, as well as temperature distributions on the fuel surface, the maximum flame temperatures and the flame zone length.

## 1. Introduction

Reinforced polymer composite materials (RPCM) are widely used in different industries, including the aviation industry. Modern requirements for the aircraft construction materials force researchers to consider ways of reducing flammability of polymer composites since these materials may participate in the reactions of exothermal oxidation and are fire-hazardous and toxic, which causes a human health hazard in the event of aircraft accidents. Glass-fiber-reinforced epoxy resin (GFRER) is one of the most promising fire-resistant construction materials used in the aircraft industry. Understanding the mechanism of ignition and burning of such composite materials, comprehensive experimental studies of the process of their combustion and developing respective models capable of predicting their behavior in different fire scenarios are important objectives for combustion science and for fire safety. Flame spread over solid fuels has been intensely studied over many decades [1,2,3]. A significant part of these works is devoted to the study of nonreinforced polymer materials, and, in particular, to the study of downward flame spread over polymethyl methacrylate (PMMA).

In the works by Bhattacharjee et al. [2], opposed-flow flame spread over thermally thin and thermally thick PMMA was investigated. The thermal regime of downward flame spread over PMMA in an oxygen–nitrogen environment in normal gravity was revisited experimentally, computationally and analytically [4]. 

Fiber-reinforced plastics are an important class of fire-resistant construction materials, which are investigated herein. Fiber reinforcement was found to influence the combustion mechanism and to act as a barrier for the heat from the flame and to prevent migration of the matrix degradation products [5]. Opposed-flow and buoyant-flow flame spread over carbon-fiber-reinforced plastic (CFRP) under variable flow velocity and oxygen concentration was investigated [6,7]. It was revealed that a change in the orientation of the carbon fibers caused thermal anisotropy, resulting in the differences in the values of the oxygen concentration limit and the flame spread rate [7]. To predict the behavior of flame spread over carbon plastic sheets, a simplified model of flame spread was developed, which included condensed-phase heat transfer. However, the authors did not develop a numerical model of flame spread for CFRP. Earlier, the thermal mechanical properties of glass-fiber-reinforced epoxy composites at elevated temperatures were investigated [8,9]. Using TGA, the kinetic parameters of thermal decomposition of glass-fiber-reinforced composites were found. Reinforcing caused anisotropy of thermal conductivity of the composite polymeric material. Thermal conductivity of a glass-fiber-reinforced plastic (GFRP) was measured in the range of temperatures from 20 to 80 K and was found to be 0.1–0.3 W/(m·K) [10]. Thermal conductivity of GFRP along the fiber direction was found to be approximately 10% lower than in the normal direction of fiber orientation. The flame retardancy behavior or fire performance of a composite material can be improved by reducing the flammability of the matrix and the reinforcing agent and by providing protective coating around the core composites [11]. Results are presented on the influence of flame retardants on the flammability of epoxy resin [8,9,12,13,14,15,16]. Pereira and Martins [5] produced an overview of the effects of nanoparticles, namely clays and carbon nanotubes, as well as different flame retardants, on the flammability of fiber-reinforced polymer composites. Meanwhile, a few works on fire-resistant glass-fiber-reinforced epoxy resins have been reported [8,9,12,17,18]. These works are devoted to the reduction in the flammability of GFRP and are limited to LOI, UL-94 and cone calorimeter tests, as well as to the determination of mechanical, thermal, physical and other properties and to the investigation of the effect of the addition of flame retardant on these parameters. At the same time, experimental studies and numerical simulation of flame propagation over glass-reinforced plastics, as contrasted to the case for nonreinforced polymers, have not been found in the literature.

To predict the behavior of flame spread over polymer surfaces, a number of numerical models were developed and tested for comparison with the experimental data. The up-to-date level of mathematical formulation [2,19,20,21] is based on the following approaches: coupled heat and mass transfer between flame and solid fuel, finite rates of chemical reactions for gas-phase combustion and solid fuel pyrolysis and consideration of gas and surface radiation. However, the developed coupled models are applied, as a rule, to simple homogeneous systems, such as flame spread over PMMA, with a simple mechanism of chemical decomposition. The aim of this work was to fill this gap and to experimentally study flame propagation over vertically oriented GFRER slabs of variable thickness in a counterflow of a N_2_/O_2_ mixture with different oxygen concentrations, as well as to develop a coupled combustion model to be validated with the experiment. The experimental and numerical data obtained under counterflow conditions are important from the perspective of justifying the limiting oxygen index test.

## 2. Experimental Section

### 2.1. Materials

In this work, glass-fiber-reinforced epoxy resin (GFRER) slabs 25 mm wide, 100 mm long, 0.3 mm and 1 mm thick were used. The slabs were prepared from prepreg based on T-15 (P)-76(92) fabric. The binder content in the prepreg was 35%. The binder consisted of 98 parts by weight of ED-22 resin, 2 parts by weight of active diluent E-181 and 5 parts by weight of curing agent #9. The slabs were made by vacuum forming with the following curing mode: 90 °C–2 h, 135 °C–2 h. In the experiments, the slab was placed in the sample holder; therefore, the width of the part of the specimen open to the flame was 20 mm (the total width of the sample was 25 mm). Samples 1.2 mm thick were prepared using the same technology but by using DYHARD Dicyandiamide OKD 100S curing agent and DYHARD UR400 accelerator. The thermophysical properties of the fiberglass samples, such as thermal diffusivity and heat capacity, were determined using laser flash methods (the flash method) on an automated setup LFA-427 by NETZSCH (Selb, Germany) and a DSC 404 F1 differential scanning calorimetry setup in the temperature range of 300–428 K in a static atmosphere of high-purity argon. The LOI for GFRER samples was 23.4%, while for epoxy resin, the LOI was 21%.

### 2.2. Thermal Degradation Analysis

Thermal decomposition of the samples was studied using thermogravimetric analysis (TGA). Pieces of GFRER slabs weighing 3–4 mg were placed in an aluminum crucible using a synchronous TG/DSC analyzer STA 409 PC (Netzsch) in a helium flow with volumetric velocity of 27 cm^3^/min (NTP). The samples were heated from 30 °C to 550 °C at the heating rates of 10, 20 and 30 K/min. All the experiments were repeated at least 2 times.

### 2.3. Flame Spread Experiments

**Figure 1:**
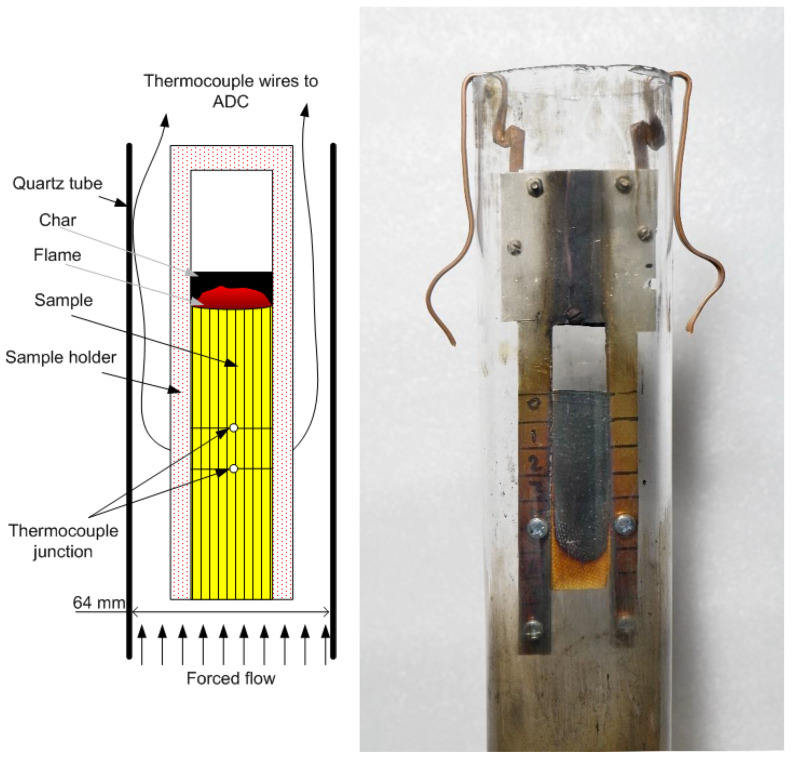
The schematic of the experimental setup (**left**) and its photo (**right**).

The GFRER slabs were inserted into a thin metal frame (sample holder, Figure 1) to prevent flame spread along the side surfaces, while the width of the open surface of the sample (over which the flame propagated) was 20 mm. The sample and the frame were marked with a step of 10 mm to measure the ROS from the video recording of the experiments with a FujiFilm x-A20 camcorder (the shooting frequency was 30 frames per second).

The experimental setup for studying downward flame propagation is shown in Figure 1. The sample was suspended in a cylindrical transparent quartz tube with a diameter of 64 mm and a length of 45 cm using a duralumin holder. Using MKS flow controllers, a mixture of N_2_ and O_2_ at various concentrations (25 v% O_2_–40 v% O_2_) was fed into the tube through polyethylene hoses. A honeycomb, a foam rubber flow equalizer, was installed in the pipe at the inlet. For all types of samples and oxygen concentrations, the flow rate was fixed during the experiment at 4 cm/s. The same flow rate was used in the LOI test. For certain cases, we varied the flow rate in the range of 2–19 cm/s. The sample was ignited from above using a propane–butane burner after turning on the opposed oxidizer flow.

The thermocouple on the slab surface was made from Pt-Pt Rh10% wire with a diameter of 50 μm. It was installed into a dip with the depth of 0.1 mm in the center of the sample and fixed with epoxy resin. Another similar thermocouple was installed at a distance of 10 mm from the first one (in the center of the sample) at a height of 1.2 mm from the slab surface to measure the temperature in the flame. The thermocouple ends were connected to wires attached to the sample holder. The wires were connected to an E14–140M multichannel ADC. The thermocouples’ reading speed was 100 Hz (at a flame propagation rate of 1 mm/s, which corresponded to the spatial resolution of 10 μm). Correction of the thermocouple measurements for radiation was calculated by using the formula proposed in [22]. All the experiments were repeated at least 3 times.

## 3. Numerical Section

### 3.1. Formulation

The developed mathematical model involved coupled heat and mass transfer between gas-phase combustion in flame and solid fuel pyrolysis, which provided a proper description of self-sustained flame propagation. Governing equations for the gas-phase were of a generally accepted statement, as follows [19,21,23,24,25]:

(1) $$\frac{\partial \rho }{\partial t}+\frac{\partial \rho u_{j}}{\partial x_{j}}=0$$

(2) $$\rho \frac{\partial u_{i}}{\partial t}+\rho u_{j}\frac{\partial u_{i}}{\partial x_{j}}=-\frac{\partial p}{\partial x_{i}}+\frac{\partial }{\partial x_{j}}\mu \frac{\partial u_{i}}{\partial x_{j}}+\left(\rho_{a}-\rho \right)g_{i}$$

(3) $$\rho C\frac{\partial T}{\partial t}+\rho u_{j}C\frac{\partial T}{\partial x_{j}}=\frac{\partial }{\partial x_{j}}\lambda \frac{\partial T}{\partial x_{j}}+\rho WQ-\frac{\partial q_{j}^{r}}{\partial x_{j}}$$

(4) $$\rho \frac{\partial Y_{F}}{\partial t}+\rho u_{j}\frac{\partial Y_{F}}{\partial x_{j}}=\frac{\partial }{\partial x_{j}}\rho D\frac{\partial Y_{F}}{\partial x_{j}}-\rho W$$

(5) $$\rho \frac{\partial Y_{O}}{\partial t}+\rho u_{j}\frac{\partial Y_{O}}{\partial x_{j}}=\frac{\partial }{\partial x_{j}}\rho D\frac{\partial Y_{O}}{\partial x_{j}}-\rho \nu_{O}W$$

(6) $$\rho \frac{\partial Y_{P}}{\partial t}+\rho u_{j}\frac{\partial Y_{P}}{\partial x_{j}}=\frac{\partial }{\partial x_{j}}\rho D\frac{\partial Y_{P}}{\partial x_{j}}+\left(1+\nu_{O}\right)\rho W$$

(7) ρ=p/RT

Here, $x_{i}=\left\{x,y\right\}$, $u_{i}=\left\{u,v\right\}$ and $g_{i}=\left\{g,0\right\}$. Gas-phase combustion in flame is described by a one-step macroscopic reaction:

(8) $$F+\nu_{O}O+I\to \left(1+\nu_{O}\right)P+I$$

(9) $$W=kY_{F}Y_{O}\exp\left(-E/R_{0}T\right)$$

Unlike the homogeneous polymeric fuels, such as PMMA [21,23] or polyformaldehyde [24], used in the previous studies, the solid fuel considered here was a composite of combustible organic binder (epoxy resin) reinforced with noncombustible glass fiber fabric. Therefore, the equation for solid fuel heat transfer was modified according to this behavior:

(10) $$\rho_{s}C_{s}\frac{\partial T_{s}}{\partial t}=\frac{\partial }{\partial x_{j}}\lambda_{s}^{j}\frac{\partial T_{s}}{\partial x_{j}}+\eta_{b}^{0}\rho_{b}Q_{b}W_{b}$$

The pyrolysis reaction of the combustible component was expressed as

(11) $$W_{b}={\left(1-\alpha \right)}^{n}A\exp\left(-E_{b}/R_{0}T_{s}\right)$$

and the conversion degree (varying from 0 to 1) was defined as

(12) $$\frac{d\alpha }{dt}=W_{b}$$

The overall density of solid material was defined as

(13) $$\rho_{s}=\eta_{b}^{0}\left(1-\alpha \right)\rho_{b}+\left(1-\eta_{b}^{0}\right)\rho_{f}$$

The mass rate of the gaseous pyrolysis product from the burning surface was expressed as

(14) $${\dot{m}}_{b}(x)=\eta_{b}^{0}\rho_{b}\int_{0}^{h}W_{b}dy$$

The boundary conditions for the set of Equations (1)–(6) and (10) were of a general type [19,20].

### 3.2. Input Data

The density of epoxy resin was $\rho_{b}$ = 1165 kg/m^3^ and the density of the glass fiber fabric was $\rho_{f}$ = 1670 kg/m^3^. From the data of sample manufacturing the binder mass fraction was $\gamma_{b}^{0}$ = 0.35 corresponding to volume fraction $\eta_{b}^{0}=\frac{\gamma_{b}^{0}/\rho_{b}}{\left[\gamma_{b}^{0}/\rho_{b}+\left(1-\gamma_{b}^{0}\right)/\rho_{f}\right]}$ = 0.44, and initial density of the composite was $\rho_{s}=\eta_{b}^{0}\rho_{b}+\left(1-\eta_{b}^{0}\right)\rho_{f}$ = 1450 kg/m^3^, which further decreased during the pyrolysis reaction according to Equation (13). Our experimental study showed the GFRER thermal conductivity in the normal direction of the fiber fabric laminates to be $\lambda_{s}^{y}$ = 0.25 W/m/K. This value stands in agreement with previous data [8]. The results of the measurements [10] show that the GFRER thermal conductivity in the direction along the fibers (for the present case—toward the flame spread) was about 15% less due to the lower thermal conductivity of glass fiber, compared to the epoxy resin. Thus, the value $\lambda_{s}^{x}$ = 0.20 W/m/K was assigned for calculations. The GFRER specific heat capacity at the temperature close to the burning surface surroundings, according to our experimental results, was set to $C_{s}$ = 1400 J/kg/K. The kinetic and thermal parameters of the pyrolysis reaction were determined in the experiment described below.

It has been shown [26] that gaseous fuel in the GFRER flame consists of low-molecular gases, such as methane, carbon monoxide, hydrogen and others. Therefore, the previously approved [21,23,25] kinetic parameters of the gas-phase combustion reaction were applied here: activation energy of E = 90 kJ/mol and pre-exponential factor of $k=10^{11}$ 1/s. The heat release of GFRER combustion was found to be Q = 25.5 MJ/kg, according to the measurements [27].

**Figure 2:**
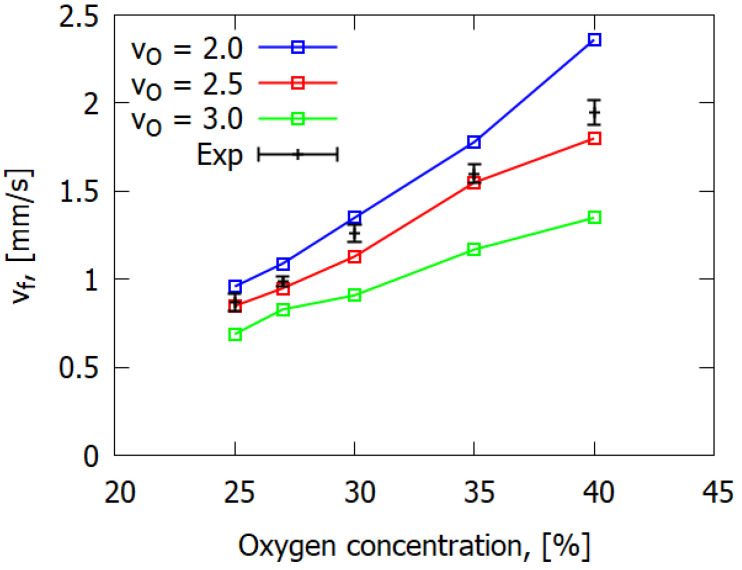
The effect of stoichiometric coefficient on flame spread rate; the number of the calculated curve corresponds to the value of the stoichiometric coefficient.

For previously studied polymeric materials, such as PMMA [21,23,25] and POM [24], the gaseous pyrolysis product of which has a rather simple chemical structure (a monomer), the value of stoichiometric coefficient $\nu_{O}$ in Equations (5) and (6) is known. On the contrary, the composite materials considered here such as GFRER produce a variety of complex chemical compositions under thermal degradation, and there is definitive uncertainty in the assignment of the stoichiometric coefficient. So, this value was set to be a parameter that would be chosen in the test run of calculations aimed to achieve agreement with the experimental data on the flame spread rate (presented at Figure 2). Finally, the stoichiometric coefficient $\nu_{O}$ was assigned as 2.5.

## 4. Results and Discussion

### 4.1. Pyrolysis Kinetics

**Figure 3:**
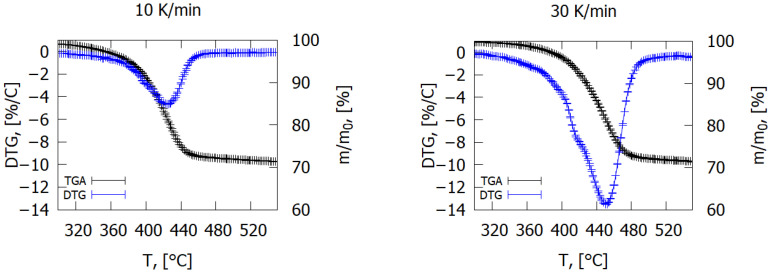
TGA data of GFRER (with curing agent #9) in inert medium.

Thermogravimetric (TGA) and differential thermogravimetric (DTG) data for the GFRER with curing agent #9 in an inert (He) medium at heating rates of 10 and 30 K/min are shown in Figure 3. At a heating rate of 30 K/min, two stages of thermal decomposition were observed. The first, less-noticeable stage was observed at a lower temperature than the second stage, in which the maximum decomposition rate was observed. The fraction of the residue (char) at 550 °C was 71.4%.

Assuming that the pyrolysis reaction occurs in one stage and is of the first order, from the data in Figure 3, the kinetic parameters of pyrolysis were obtained using the established method [28]. The obtained kinetic parameters for GFRER with curing agent # 9 were: *n* = 1, *E* = 160.8 kJ/mol and *A* = 8.5 × 10^9^ 1/s. Kinetic parameters for GFRER with the OKD 100S curing agent were as follows: *n* = 1, *E* = 112 kJ/mol and *A* = 3.8 × 10^6^ 1/s. The pyrolysis rate constant of GFRER with a different type of curing agent in the Arrhenius-type plot is shown in Figure S1 (Supplementary Materials). The use of the OKD 100S curing agent resulted in a higher pyrolysis rate of the GFRER slabs. The data obtained were used in the GFRER combustion simulation. Unlike thermal decomposition of PMMA, that of GFRER produces carbon residue, char. 

### 4.2. Dependence of the Flame Propagation Rate over GFRER on the Oxygen Concentration in the Gas Flow and on the Slab Thickness

**Figure 4:**
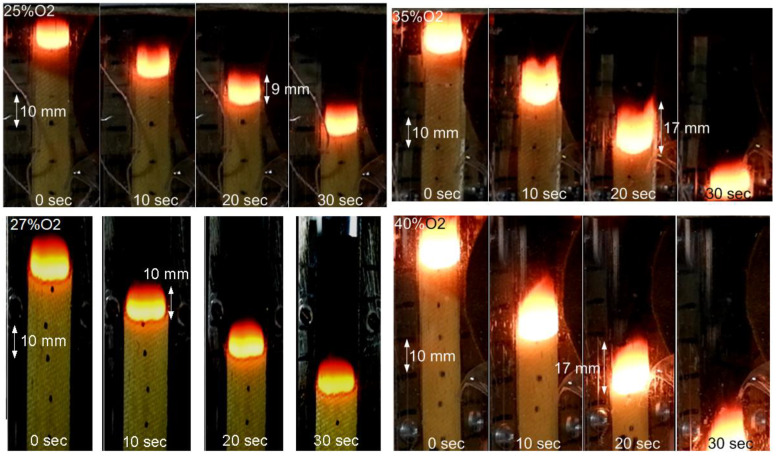
Photos of flame propagation over 0.3 mm-thick GFRER slabs at oxygen concentrations varying from 25% to 40%.

Figure 4 shows photographs of flame propagation over the 0.3 mm-thick GFRER slabs at different moments in time. No dripping was observed when the flame spread over the samples. The moment when the flame crossed the first mark was selected as 0 s (after passing 10 mm from the ignition site). As the oxygen concentration increased from 25% to 40%, the ROS increased from 0.87 mm/s to 1.95 mm/s, and the combustion zone length (CZL) increased from 9 mm to 17 mm. A similar effect of O_2_ concentration on the ROS and the flame size was observed [7] for the flame spread over carbon-fiber-reinforced epoxy resin in the opposed-flow conditions. However, there are no data on the effect of the CFRP slab thickness on the combustion parameters [7].

**Figure 5:**
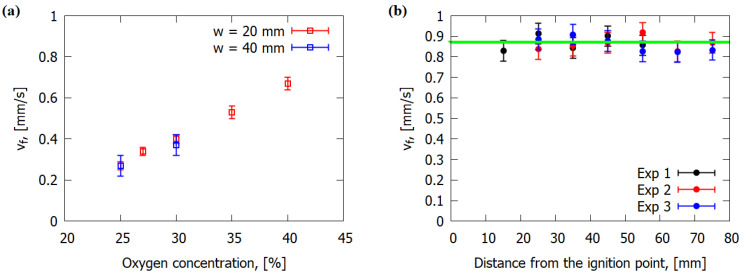
The influence of the slab width (*w*) on the measured ROS over 1.2 mm-thick GFRER samples (**a**). Dependence of ROS on the distance from the ignition point for 0.3 mm-thick sample, 25% O_2_ (**b**). Dots of different colors correspond to different runs of the experiment. The green line corresponds to the mean value of *v_f_*.

The effect of the slab width on the ROS was studied. Figure 5a shows the ROS versus oxygen concentration for 20- and 40 mm-wide samples. It can be seen that an increase in the slab width from 20 to 40 mm did not affect the ROS. When the sample side surfaces were inhibited by the noncombustible holder, the flat flame front was formed for the samples of certainly large width so that the ROS was not affected by sidewalls. Here, the width of 20 mm was found to be sufficient (as a minimum), ensuring the flat-flame-spread mode. Such an effect supports the validity of the two-dimensional numerical model presented in Section 3 in Equations (1)–(6) and (10). Therefore, all the data below were obtained for samples 20 mm wide. In Figure 5b, the dependence of the ROS (*v_f_*) on the distance from the ignition point is shown. The values of the ROS for three experiments were consistent in all repeated procedures. Figure 5b indicates the stationary mode for the rate of flame spread (when combustion is stable) appears shortly after the ignition. The ROS did not change when the direction in which the sample burned (towards the wind) was changed by 90°. In other words, the orientation of the reinforcement did not affect the ROS (but there was influence from the orientation along and perpendicular to the fibers). In the case of CFRP [6,7], the behavior of ROS was significantly different—a significant influence of the direction in which the sample was burning was observed. This is due to the dependence of thermal conductivity on the fibers’ orientation.

**Figure 6:**
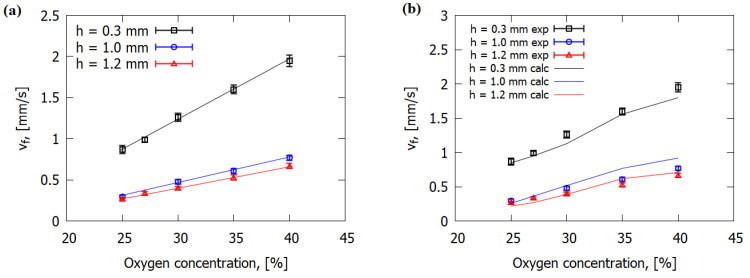
Dependence of the rate of flame propagation over GFRER on the oxygen concentration in the flow. Experiment and linear approximation (**a**); experiment and calculation (**b**).

Figure 6a shows that the experimental ROS was directly proportional to oxygen concentration, with the slope of the ROS versus O_2_ decreasing with increasing sample thickness. The increase in oxygen concentration led to the increase in the rate of the gas-phase combustion reaction defined in Equation (9), so that heat release rose in the flame zone, which, in turn, resulted in the increase in the heat flux on the solid fuel surface, which led to the increase in the ROS. At an oxygen concentration of less than 25%, the samples did not burn. It follows from Figure 6b that the simulation results are in good agreement with the experimental data, although they demonstrate certain deviation from the linear dependence for 1 mm-thick samples at 35% O_2_. The experimental error did not exceed 10%.

**Figure 7:**
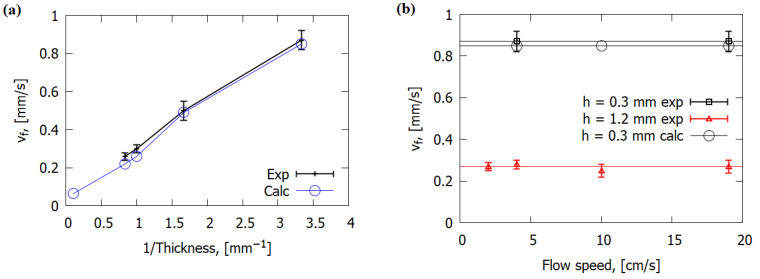
Dependence of the ROS over GFRER slabs on the slab thickness (**a**) and on the gas flow rate (**b**) at 25% O_2_.

The simulation results and the experimental data show that ROS depended inversely on the slab thickness (Figure 7a), similarly to nonreinforced polymers [2,19,29]. With the increase in thickness of sample, a greater amount of energy was consumed by inert heating and thermal degradation of solid fuel, which resulted in the decrease in the ROS.

**Figure 8:**
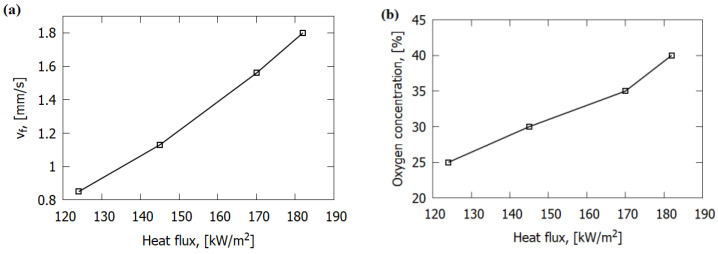
Dependence of the maximum calculated heat flux from the flame to the surface of the 0.3 mm-thick sample on the ROS (**a**) and on O_2_ concentration (**b**).

The experimentally measured ROS over GFRER, as well as the calculated one, did not depend on the flow velocity in the range of 2–19 cm/s (Figure 7b), which testifies to the thermal regime of flame propagation [4]. The calculation results show that the buoyancy velocity of the flame was in the order of 40 cm/s, which is higher than the forced convection velocity in the investigated range (up to 20 cm/s). Thus, the downward flame spread behavior under certain natural convection conditions is not affected by forced flow, unless its velocity exceeds the buoyancy effect. It can be seen that the ROS was close for samples with thicknesses of 1 and 1.2 mm, with different pyrolysis kinetics (Figure S2 in the Supplementary Materials). This is consistent with the results of the numerical study on the sensitivity of the ROS to pyrolysis kinetics, according to which an increase in the pre-exponential factor of the pyrolysis rate constant by a factor of 2–3 changed the ROS by only 15–20%. In addition, the calculated ROS (and thus the fraction of O_2_ in the gas flow) was directly proportional to the calculated maximum heat flux from the flame to the fuel (Figure 8).

### 4.3. Thermal Flame Structure

**Figure 9:**
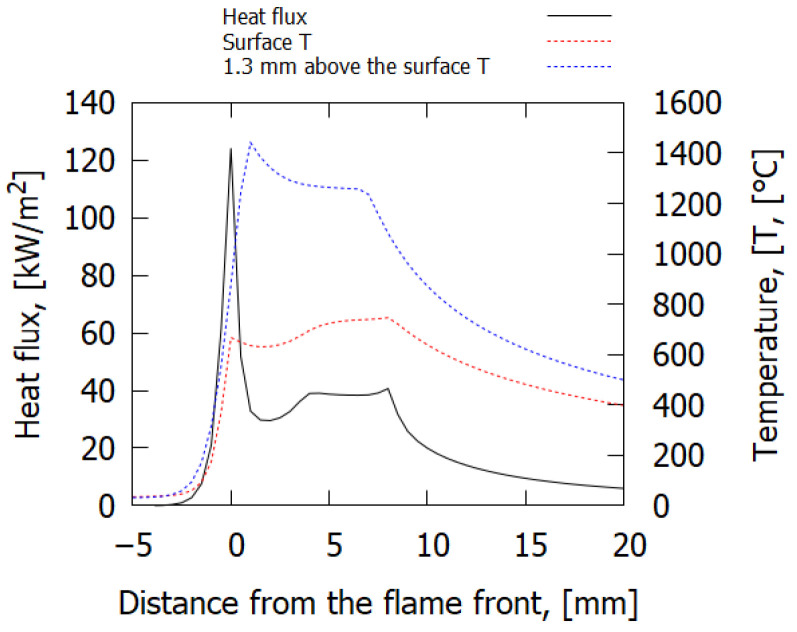
The calculated temperature profiles in the condensed and in the gas phase (at a height of 1.3 mm above the surface) and the heat flux profile for a 0.3 mm-thick sample at 25% O_2_.

In Figure 9, the calculated temperature profiles in the condensed phase and in the gas phase (at a height of 1.3 mm above the surface) and the heat flux profile for a 0.3 mm-thick sample at 25% O_2_ are presented. The maximum value of the heat flux with an accuracy of 0.5 mm coincided with the first maximum of the surface temperature (the beginning of the pyrolysis zone) and corresponded to the position of the flame front. The maximum temperature in the flame (at the height of 1.3 mm above the surface) was reached at the distance of 1 mm after the maximum surface temperature and corresponded to 1440 °C.

**Figure 10:**
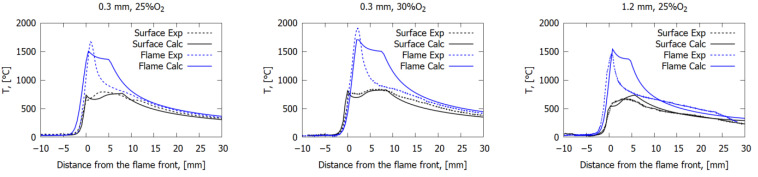
Temperature profiles in the flame (1.2 mm above the surface) and on the fiberglass plastic surface.

Figure 10 shows a comparison of the calculated and measured temperature profiles in the flame (at a height of 1.2 mm above the surface) and on the fiberglass plastic surface as the function of the distance from the flame front. Good agreement was observed between the model and the experiment for the surface temperature profiles both for the length of the combustion zone and for the maximum temperature. It can be seen that after reaching the maximum, the measured temperature profiles differed from the calculated ones, which is associated with the deposition of soot on the thermocouple. Because of this, the second temperature peak at a height of 1.2 mm was not observed in the experiment. As the oxygen concentration in the gas flow increased, the maximum value of the temperature in the flame increased both in the model and in the experiment. The effect of oxygen concentration and slab thickness on the maximum surface temperature and flame temperature (at a height of 1.2 mm) was relatively small (~15–20%).

**Figure 11:**
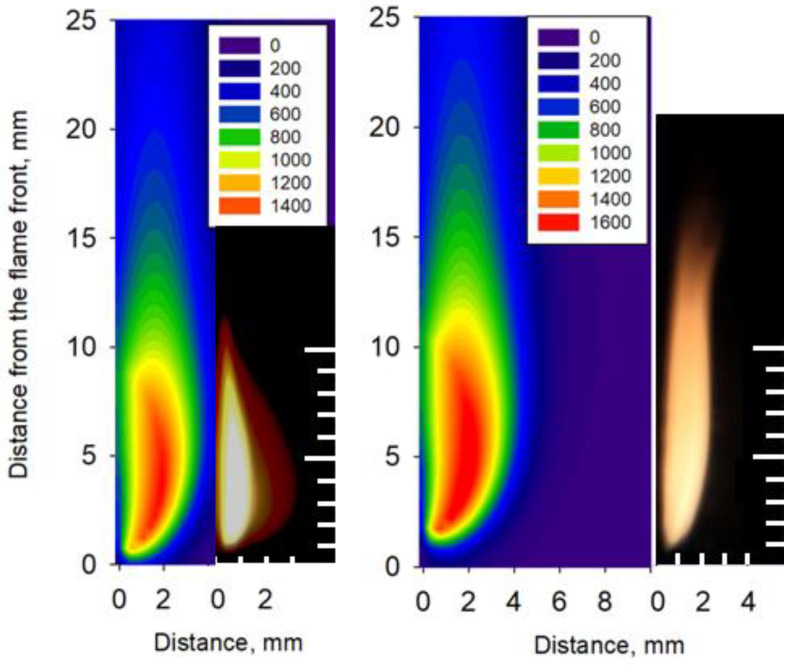
Comparison of the calculated temperature field and the photograph of the flame during the experiment. h = 0.3 mm, 25% O_2_ (**left**) and 30% O_2_ (**right**).

The solid-phase preheating length (the zone between the section where the temperature starts to rise and the nearest maximum of the surface temperature, as shown in Figure 10) for 0.3 mm-thick GFRER at 25% O_2_ was ~5 mm, which was less than the length of the combustion zone (~8–9 mm). In the case of CFRP, the length of the preheat zone was about twice the length of the flame [7]. Thus, it can be concluded that preheating in the condensed phase of fiberglass, in contrast to CFRP, does not affect the flame propagation. Figure 11 shows a comparison of a photograph of the flame in the experiment with the temperature field calculated using the model. It can be seen that the size of the luminous zone in the photograph is in good agreement with the calculated high-temperature region. In the case of 25% O_2_, the flame height was about 2–3 mm and about 4 mm in the case of 30% O_2_. The length of the combustion zone was ~8 mm and ~11 mm at 25% O_2_ and 30% O_2_, respectively, which is consistent with Figure 4 and Figure 10.

### 4.4. Sensitivity Analysis of the Model

**Figure 12:**
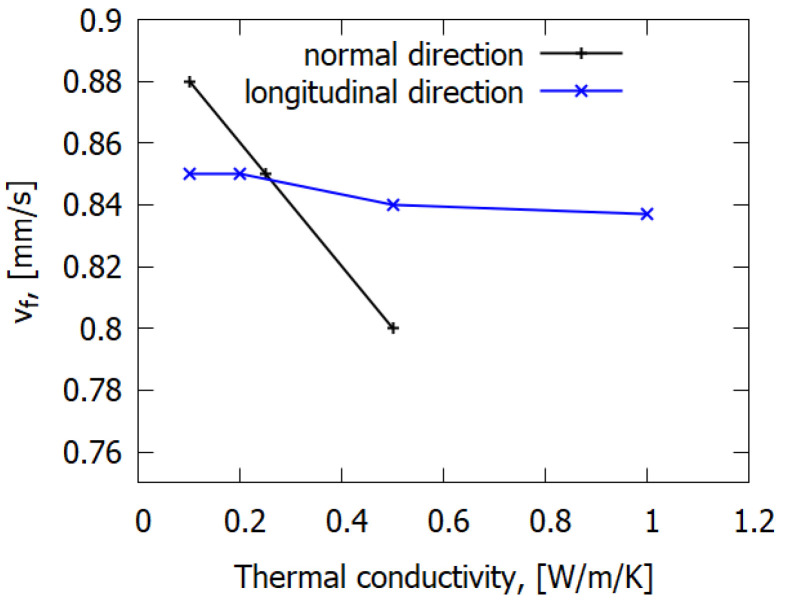
The effect of thermal conductivity in the longitudinal direction and in the normal direction on the flame spread rate. h = 0.3 mm, 25% O_2_.

Sensitivity analysis of the model was required to improve the understanding of the mechanism of flame propagation over solid fuel. As mentioned above, the presented model is able to reasonably predict the main trends of the dependences of the ROS and temperature distribution upon sample thickness, forced flow rate and oxygen concentration for the considered reinforced material. Such an analysis of the model has been carried out on the effect of solid fuel’s thermal conductivity. In contrast to CFRP [7], for which the thermal conductivity of the reinforcing fiber is up to 1000 times greater than that of the binder, such parameters for the present material, GFRER, have comparable values both for the binder and the fibers (in fact, that of the latter is even less than that of former). Thus, the simulation data (Figure 12) show that the ROS did not noticeably depend on the overall effective thermal conductivity of the fiberglass fabric along the flame spread direction. In contrast, the ROS decreased as thermal conductivity rose in the direction normal to the solid fuel’s surface (Figure 12). Heat transfer by conduction depends on the temperature gradient, the thermal conductivity of the material and the cross section area. Considering the flame spread over the solid material, the cross section area for heat transferred in the direction perpendicular to the sample’s surface was much higher than the cross section area (a thin area in the vicinity of the surface with a high temperature of the solid material) for heat transferred in the direction along the slab surface. The more heat that was supplied from the flame to the solid dissipates inside the sample, the less heat was available for thermal degradation, followed by the release of combustible gas. Thus, flame spread behavior was mainly determined by thermal conductivity in the direction perpendicular to the surface of the sample and was almost independent of the thermal conductivity in the direction parallel to the sample’s surface.

## 5. Conclusions

A comprehensive experimental and numerical study of downward flame spread over slabs of glass-fiber-reinforced epoxy resin (GFRER) under the small opposed flow of a N_2_/O_2_ mixture with varied oxygen concentrations and differing sample thicknesses has been carried out. It was found that the rate of flame spread over GFRER, which linearly depended on the oxygen concentration, was inversely proportional to the thickness of the slab and did not depend on the direction of flame propagation along the sample at the mixture flow rate in the range of 2–19 cm/s. It was found that the increase in oxygen concentration resulted in an increase in the ROS, heat flux from the flame to the surface of GFRER and flame temperature. An increase in the slabs’ thickness resulted in a decrease in the ROS. At the same time, the change in the velocity of the oxidizing flow did not affect the ROS, and the change in the kinetics of GFRER pyrolysis only slightly affected the ROS. Numerical modeling was carried out on the basis of the coupled ‘gas–solid’ model of heat and mass transfer using a modification of the OpenFOAM open-source code. The model involved one-step reactions for combustion and pyrolysis. The developed numerical model of flame propagation over GFRER predicted with good accuracy the temperature distributions on the solid fuel burning surface, maximum temperatures in the flame, the length of the combustion zone and the ROS at varied oxygen concentrations and sample thicknesses.

The sensitivity analysis of the model showed that the thermal conductivity in the normal direction of the slab had a greater effect on the ROS than the longitudinal thermal conductivity of the GFRER in the flame propagation direction. It was also found that the kinetics of GFRER pyrolysis had little effect on the ROS. These facts also indicate that in the case of fiberglass, the ROS is mainly determined by the heat transfer through the gas-phase toward the flame spread, while in the case of carbon-fiber-reinforced plastic, the ROS is determined by the solid fuel heat transfer along the fibers. Thus, heat transfer from the flame to the GFRER surface has been determined to be the main mechanism of downward flame spread over GFRER.

The data obtained are valuable for understanding the mechanism of polymer combustion for fire safety and may be used for the numerical modeling of fire spread over GFRER in other scenarios, for example, for upward and horizontal fire spread.

## Supplementary Materials

The following supporting information can be downloaded at: www.mdpi.com/article/10.3390/polym14050911/s1, Figure S1: The GFRER pyrolysis rate constant with different curing agent types in the Arrhenius plot. Figure S2: The effect of the pre-exponential factor of the pyrolysis reaction on the flame spread rate. Black–experiment, red–calculation.

## Abbreviations

CFRP	carbon-fiber-reinforced polymer
GFRER	glass-fiber-reinforced epoxy resin
LOI	limiting oxygen index
ROS	rate of flame spread
TGA	thermogravimetric analysis
DTG	differential thermogravimetric analysis
**Nomenclature**	
*A*	pyrolysis preexponential factor [s^−1^]
*C*	specific heat capacity [J/kg/K]
D	diffusion coefficient [m^2^/s]
E	activation energy [J/mol]
g	gravity acceleration [m/s^2^]
h	sample thickness [mm]
k	gas-phase preexponential factor [s^−1^]
M	molar mass [g/mol]
$\dot{m}$	mass burning rate [g/s]
n	pyrolysis reaction order [-]
p	pressure [Pa]
Q	heat release [J/kg]
q	heat flux [W/m^2^]
R	specific gas constant [J/kg/K]
$R_{0}$	universal gas constant [J/mol/K]
T	temperature [K]
t	time [s]
u	velocity [m/s]
$v_{f}$	flame spread rate [mm/s]
W	reaction rate [s^−1^]
w	width
x	coordinate along fuel surface [m]
Y	gas component mass fraction [-]
y	coordinate normal to fuel surface [m]
**Greek symbols**	
α	conversion degree [-]
γ	solid component mass fraction [-]
δ	burnout degree [-]
η	solid component volume fraction [-]
λ	thermal conductivity [W/m/K]
μ	dynamic molecular viscosity [kg/m/s]
ν	stoichiometric coefficient [-]
ρ	density [kg/m^3^]
τ	half-thickness [m]
**Subscripts**	
a	ambient
b	binder
F	fuel
*f*	fiber
g	gas
I	inert component
O	oxidizer
P	product
p	pyrolysis
s	solid
**Superscripts**	
r	radiative
x	along fuel surface
y	normal to fuel surface
