# Supplementary material for: Experimental and Numerical Study of Downward Flame Spread over Glass-Fiber-Reinforced Epoxy Resin
Source: Polymers (Basel). 2022 Feb 24;14(5):911. doi: 10.3390/polym14050911 (PMC8912476; doi:10.3390/polym14050911)
Supplement: Supplementary file 1 [file polymers-14-00911-s001.zip › polymers-1584398-supplementary.pdf]

Supplemental Materials

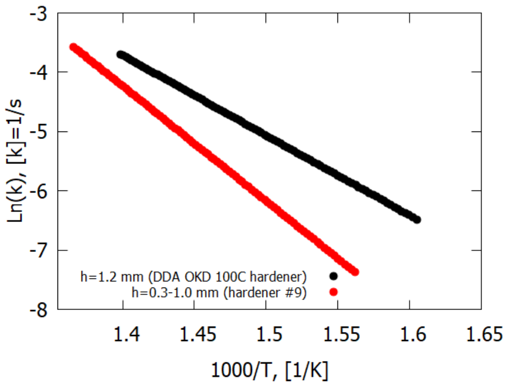

Figure S1. The GFRER pyrolysis rate constant with different curing agent types in the Arrhenius plot.

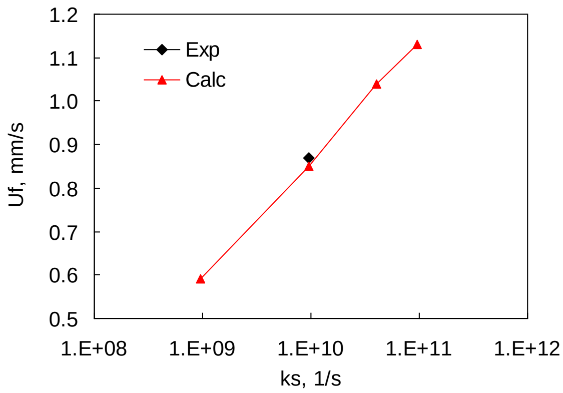

Figure S2. The effect of the pre-exponential factor of the pyrolysis reaction on the flame spread rate. Black – experiment, red – calculation.
